# Dynamic trends of ischemic heart disease mortality attributable to high low-density lipoprotein cholesterol: a joinpoint analysis and age-period-cohort analysis with predictions

**DOI:** 10.1186/s12944-024-02274-y

**Published:** 2024-09-11

**Authors:** Min Li, Beibei Wang, Lan Wang, Ling Tong, Gang Zhao, Biao Wang, Jingli Guo

**Affiliations:** 1 Department of Cardiology, Shanxi Provincial People’s Hospital, Fifth Hospital of Shanxi Medical University, Taiyuan, Shanxi 030012 China; 2 Department of Cardiology, The First People’s Hospital of Jinzhong, Jinzhong, 030602 China; 3 School of Foreign Languages, Yantai University, Yantai, Shandong 264005 China; 4 Department of Cardiology, Wenshui People’s Hospital, Wenshui, Shanxi 032100 China; 5 Medical Department, Shanghai Ashermed Medical Technology Co., Ltd, Shanghai, 200030 China

**Keywords:** Ischemic heart disease, High low-density lipoprotein cholesterol, Joinpoint analysis, Age-period-cohort analysis, Prediction analysis

## Abstract

**Aims:**

The purpose of this study was to analyze the dynamic trends of ischemic heart disease (IHD) mortality attributable to high low-density lipoprotein cholesterol (LDL-C).

**Methods:**

Data on IHD mortality attributable to high LDL-C from 1990 to 2021 were extracted from the global disease burden database. Joinpoint software was used to estimate the average annual percentage change (AAPC) in the age-standardized mortality rate (ASMR). An age‒period‒cohort model was used to analyze the impacts of age, period, and cohort on these changes. The Bayesian framework was used to predict IHD mortality attributable to high LDL-C from 2022 to 2040.

**Results:**

The overall ASMR of IHD attributable to high LDL-C decreased from 50. 479 per 100,000 people in 1990 to 32.286 per 100,000 people in 2021, and ASMR of IHD attributable to high LDL-C was higher in males than in females. The longitudinal age curves of the overall IHD mortality attributable to high LDL-C showed a monotonic upward trend, especially after 65 years of age. The period and cohort effect relative risk (RR) values of overall IHD mortality attributable to high LDL-C showed a downward trend. The overall ASMR of IHD attributable to high LDL-C is predicted to show a downward trend, and male IHD mortality attributable to high LDL-C is expected to be higher than that of females.

**Conclusion:**

This study revealed a sustained decrease in IHD mortality attributable to high LDL-C over three decades, with a continued decline expected. Despite this, gender disparities persist, with males experiencing higher mortality rates and elderly individuals remaining a vulnerable group.

## Introduction

Cardiovascular disease (CVD), especially ischemic heart disease (IHD), is the main cause of death and disability worldwide and remains the main cause of the burden of disease worldwide [1]. CVD is currently a highly valued public health issue worldwide, with IHD receiving widespread attention because of its high mortality rate. Notably, metabolic risk factors have become the main driving factor for IHD, with high low-density lipoprotein cholesterol (LDL-C) still being an important factor [2]. In the United States, medical expenses associated with IHD account for 1%-1.5% of the gross domestic product, and the expenses associated with each episode of IHD exceed $5,000 [3]. In low- and middle-income countries, the median IHD total healthcare expenditure per capita for specific national health expenditures is 10% of total healthcare expenditure [4]. The medical burden of IHD mainly consists of outpatient visits, emergency visits, prescription drug treatment, revascularization procedures, and hospitalization [5]. As a CVD, IHD is caused mainly by coronary atherosclerosis, and the formation of atherosclerotic plaques is caused mainly by the accumulation of cholesterol in the arterial wall, which narrows or occludes the coronary artery [6]. Clinical research has confirmed that high LDL-C is one of the main risk factors for CVD. Reducing high LDL-C levels through medication can reduce the risk of CVD events to some extent [7]. Therefore, early prevention and treatment of high LDL-C can effectively prevent and reduce CVD events, especially IHD.

We recognize that previous studies have begun to quantify the epidemiological trends of IHD mortality related to high LDL-C. For example, a pivotal study by Du et al. [8] demonstrated that, in 2019, IHD was the major disease attributable to high LDL-C, accounting for 86.1% of high LDL-C-related deaths. However, despite these findings, there remains a significant gap in the literature regarding the comprehensive analysis of long-term trends and future projections of IHD mortality attributable to high LDL-C on a global scale. Our study seeks to address this gap by leveraging extensive and systematic data from the Global Burden of Disease (GBD) study 2021. We aim to provide a thorough analysis that considers the long-term trends and future projections of IHD mortality attributable to high LDL-C. This analysis contributes to a more strategic approach to health planning and policy development, with the goal of mitigating the burden of high LDL-C-related IHD mortality globally.

## Materials and methods

### Data sources

The GBD 2021[9], conducted by the Institute of Health Metrics and Evaluation at the University of Washington, USA, offers a robust and timely assessment of epidemiological profiles across a vast array of diseases, injuries, and risk factors globally. Owing to its extensive global data coverage and comprehensive risk factor evaluation, GBD is particularly advantageous for this study, providing a rich dataset that spans 204 countries and regions from 1990–2021. GBD's collaborators described a detailed comprehensive methodological risk assessment for high LDL-C, defined as blood LDL-C concentrations exceeding the theoretical minimum risk exposure level, that is, 1.3 mmol/L (50 mg/dl) [10]. In this study, high LDL-C-related IHD data for males and females aged 25–95 years were selected from the GBD 2021 database from 1990 to 2021. Age-standardized mortality rate (ASMR) and number of deaths directly extracted from the GBD database and represented by the 95% confidence interval (UI).

### Joinpoint regression model

The joinpoint regression model identifies the optimal connection point by performing logarithmic linear fitting on the mortality of outcomes studied during a specific period and then divides the long-term trend of research outcomes into several continuous intervals. This study utilized a joinpoint regression model to analyze the overall, male and female trends in IHD mortality attributable to high LDL-C from 1990 to 2021. The annual percentage change (APC), average annual percentage change (AAPC) and 95% confidence interval (CI) were calculated for each group.

### Age-period-cohort model

The age‒period-cohort model considers the age effect, which reflects the natural progression of mortality risk with age, the period effect, which captures the impact of time-specific factors, such as medical advancements, on mortality rates, and the cohort effect, which accounts for generational differences in exposure to risk factors. It is mainly used to analyze the temporal trend of chronic disease mortality and predict future changes in disease burden on the basis of the Poisson distribution [11], whereas the intrinsic estimator method related to the age‒period-cohort model can solve the problem of "nonidentifiability" caused by the linear relationship between age and cohort, increasing the accuracy of the estimation results [12]. Relative risk (RR) is used to quantify the effects of age, period, and cohort, along with net and local drift values, to describe annual changes in mortality rates across the study period and within age groups.

### Prediction model

The Bayesian age‒period-cohort (BAPC) model can predict the ASMR and number of deaths. The fitting of this model usually uses the Markov chain Monte Carlo method (MCMC), which is prone to introducing complex convergence problems and may be affected by other technical problems. The integration of the integrated nested Laplace approximation (INLA) offers a solution to these problems, enhancing the model's reliability and accuracy in predictions [13].

### Statistical methods

Joinpoint regression analysis was performed via the Joinpoint Regression Program 4.7.0.0 software to calculate APC and AAPC. An APC > 0 indicates an upward trend in mortality, an APC < 0 indicates a downward trend, and an APC = 0 indicates no change in mortality. APC = AAPC represents a monotonic increase or decrease in the mortality rate [14]. The age‒period-cohort model was analyzed via a web tool developed by the National Cancer Institute of the United States via the open-source software R language [15]. The main parameters used in age‒period-cohort model analysis include net drift, local drift, longitudinal age curve, period RR, and cohort RR. The general linear model was used to test the difference in the RR slope between the period and the cohort. On the basis of the 1990–2021 time series data of global IHD mortality attributable to high LDL-C and the 60-year (1990–2040) time series data of the world population, this study uses the BAPC package in Rstudio4.2.3 to carry out Bayesian age‒period-cohort modeling and predict IHD mortality attributable to high LDL-C in the next 19 years. A *P* value less than 0.05 is considered statistically significant.

## Results

### Trends of IHD mortality attributable to high LDL-C

**Fig. 1:**
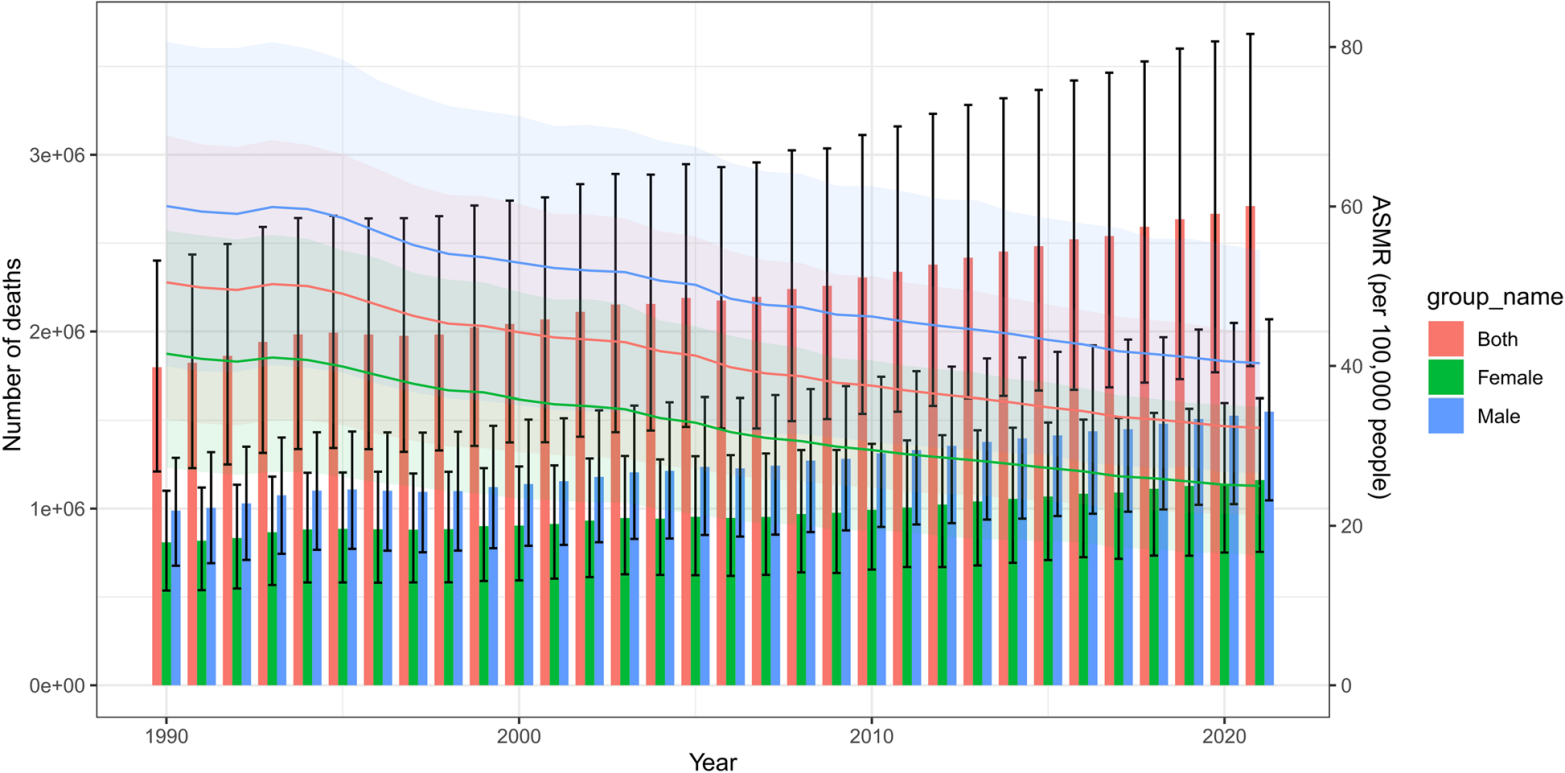
Trends in the number of deaths and the ASMR per 100,000 people for IHD attributable to high LDL-C. IHD; ischemic heart disease, ASMR, age-standardized mortality rate; LDL-C, low-density lipoprotein cholesterol

Our analysis revealed that the number of IHD deaths attributable to high LDL-C has been consistently increasing, in contrast to the ASMR, which has demonstrated a downward trajectory. The overall number of IHD deaths attributable to high LDL-C increased from 1.798 (95% UI: 1.210 to 2.401) million in 1990 to 2.710 (95% UI: 1.804 to 3.684) million in 2021. The number of IHD deaths attributable to high LDL-C in males and females tended to increase, and the number of IHD deaths attributable to high LDL-C in males was greater than that in females. The number of deaths for males increased from 0.989 (95% UI: 0.676 to 1.287) million in 1990 to 1.548 (95% UI: 1.046 to 2.069) million in 2021, and the number of deaths for females increased from 0.809 (95% UI: 0.536 to 1.101) million in 1990 to 1.161 (95% UI: 0.755 to 1.623) million in 2021. However, after age standardization according to the standard population, the overall ASMR of IHD attributable to high LDL-C decreased. The ASMR of IHD attributable to high LDL-C in males and females also showed a decreasing trend, and the ASMR in males was greater than that in females. The overall ASMR of IHD attributable to high LDL-C decreased from 50.479 (33.388 to 68.905) per 100,000 people in 1990 to 32.286 (21.353 to 44.059) per 100,000 people in 2021. The ASMR for males decreased from 60.050 (39.953 to 80.638) per 100,000 people in 1990 to 40.339 (26.657 to 54.537) per 100,000 people in 2021, and the ASMR for females decreased from 41.536 (27.303 to 56.956) per 100,000 people in 1990 to 25.007 (16.277 to 34.898) per 100,000 people in 2021 (Table 1, Fig. 1).

**Table 1:** Trends of IHD mortality attributable to high LDL-C from 1990 to 2021

	**Number of deaths ** **No.×106 (95% UI)**		**ASMR per 100,000 ** **No. (95% UI)**		**APC(%,95CI%)**	**AAPC(%,95CI%)**	**T **	*P *for AAPC
	**1990 **	**2021 **	**1990**	**2021**				
Overall(Both)	1.798(1.210,2.401)	2.710(1.804,3.684)	50.479(33.388,68.905)	32.286(21.353,44.059)		-1.430(-1.590 to -1.269)	-17.3498	＜0.001
1990-1994					-0.023(-0.506 to 0.463)			
1994-1998					**-2.465(-3.20 to -1.724)**^a^			
1998-2003					-1.082(-1.56 to -0.602)^a^			
2003-2007					-2.283(-3.03 to -1.53)^a^			
2007-2021					-1.411(-1.482 to -1.34)^a^			
Females	0.809(0.536,1.101)	1.161(0.755,1.623)	41.536(27.303,56.956)	25.007(16.277,34.898)		-1.628(-1.794 to -1.462)	-19.0479	＜0.001
1990-1994					-0.287(-0.788 to 0.216)			
1994-1998					**-2.420(-3.183 to -1.650)**^a^			
1998-2003					-1.344(-1.843 to -0.843)^a^			
2003-2007					-2.675(-3.443 to -1.901)^a^			
2007-2021					-1.582(-1.657 to -1.508)^a^			
Males	0.989(0.676,1.287)	1.548(1.046,2.069)	60.050(39.953,80.638)	40.339(26.657,54.537)		-1.270(-1.429 to -1.110)	-15.4638	＜0.001
1990-1994					0.052(-0.426 to 0.531)			
1994-1998					**-2.512(-3.243 to -1.775)**^a^			
1998-2003					-0.868(-1.345 to -0.389)^a^			
2003-2007					-1.907(-2.655 to -1.153)^a^			
2007-2021					-1.248(-1.319 to -1.176)^a^			

*IHD* Ischemic heart disease, *LDL-C *Low-density lipoprotein cholesterol, *APC* Annual percentage change, *AAPC* Average annual percentage change, *ASMR *Age-standardized mortality rate, *ASR* Age standardized rate, *CI* Confidence interval, *UI* Uncertainty interval

^a^Represents *P*＜0.05 for APC

**Fig. 2:**
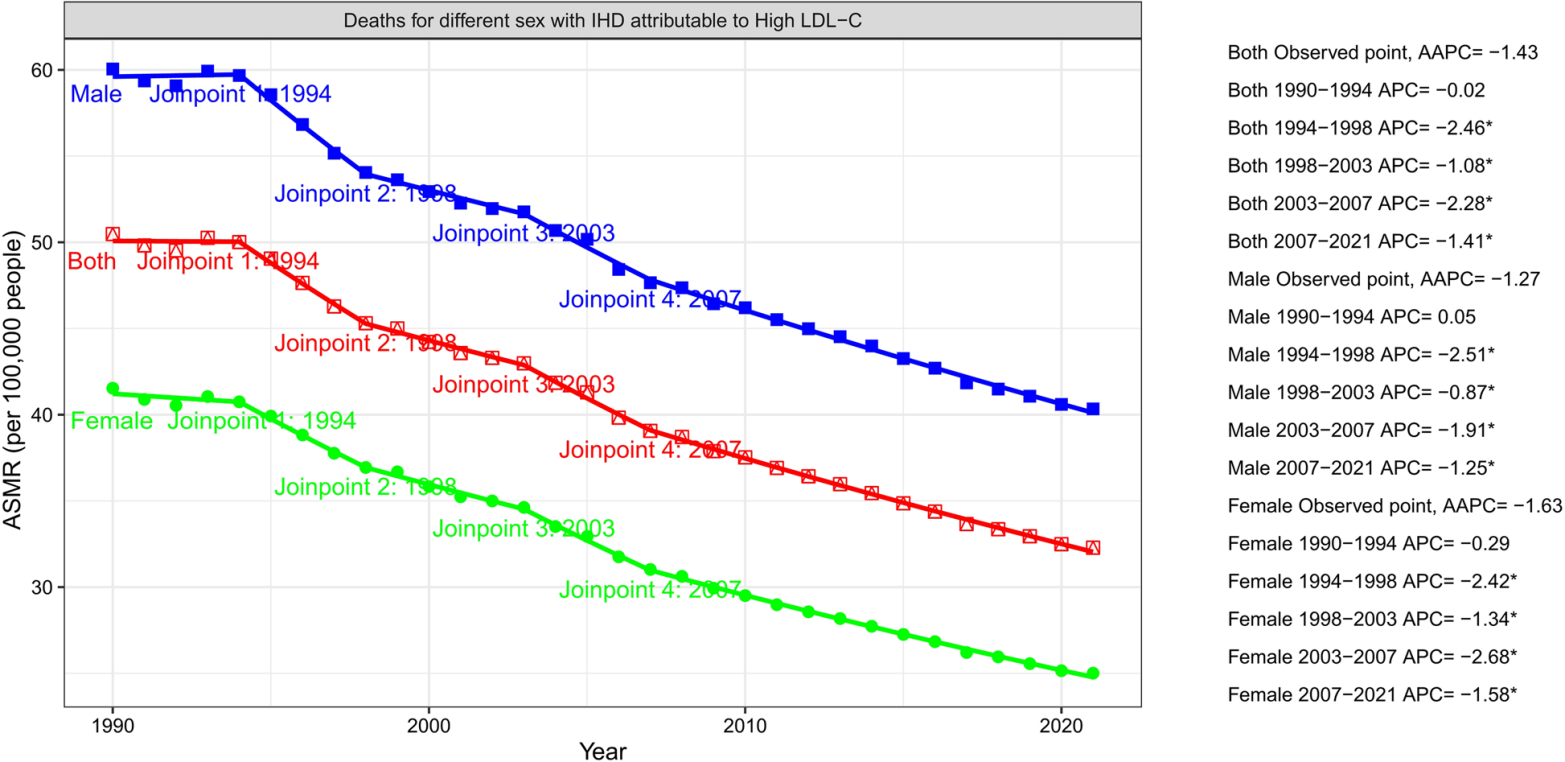
Joinpoint trend of the ASMR for IHD attributable to high LDL-C from 1990–2021. IHD, ischemic heart disease; ASMR, age-standardized mortality rate; LDL-C, low-density lipoprotein cholesterol

A joinpoint regression model revealed that the ASMR of IHD attributable to high LDL-C for males and females and the overall population showed a downward trend, and the trend changes were statistically significant (*P* < 0.05). The overall ASMR of IHD attributable to high LDL-C has an AAPC of − 1.430% (95% CI: − 1.590 to − 1.269; *P* < 0.001), the female ASMR has an AAPC of − 1.628% (95% CI: − 1.794 to − 1.462); *P* < 0.001), and the male ASMR has an AAPC of − 1.270% (− 1.429 to − 1.110; *P* < 0.001). Overall, male and female ASMRs of IHD attributable to high LDL-C decreased the fastest between 1994 and 1998, with statistical significance (*P* < 0.05). (Table 1, Fig. 2).

### Age-period-cohort model analysis of IHD mortality attributable to high LDL-C

**Fig. 3:**
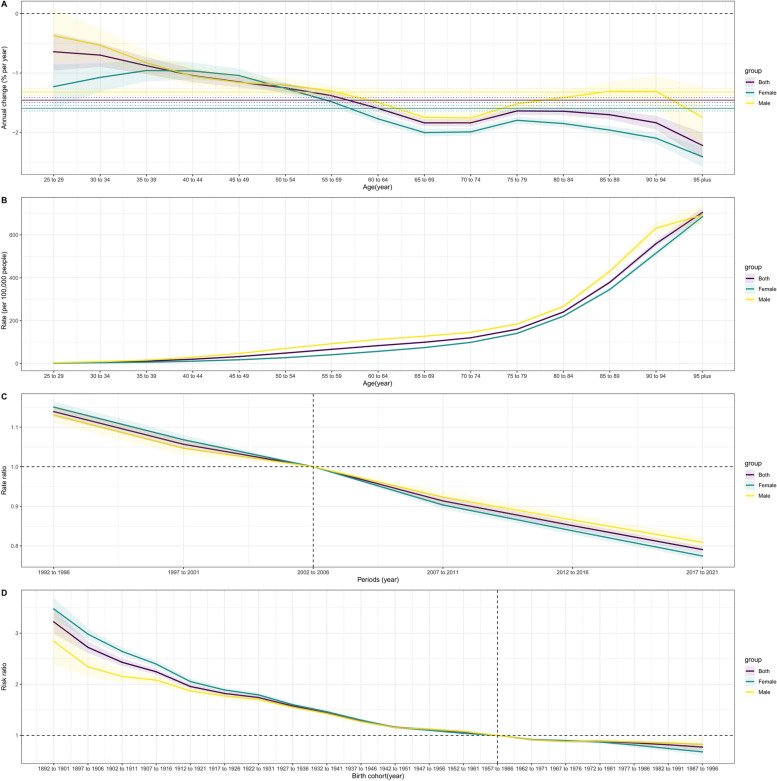
Age-period-cohort analysis of IHD mortality attributable to high LDL-C from 1990–2021. Local drift with net drift values for IHD mortality attributable to high LDL-C (**A**). Longitudinal age curves of IHD mortality attributable to high LDL-C (**B**). Period relative risk (RR) of IHD mortality attributable to high LDL-C (**C**). Cohort relative risk (RR) of IHD mortality attributable to high LDL-C (**D**). IHD, ischemic heart disease; RR, relative risk; LDL-C, low-density lipoprotein cholesterol

The overall annual percent changes (net drifts) and age-specific annual percent changes (local drifts) in IHD mortality attributable to high LDL-C are shown in Fig. 3A. The net drift value of overall IHD mortality attributable to high LDL-C was − 1.456% per year, and the net drift values were − 1.320% per year for males and − 1.592% per year for females. The local drift value of the IHD mortality attributable to high LDL-C in males and the overall population showed a downward, upward and downward trend, whereas that of females showed an upward, downward, upward and downward trend. The peak values of overall and male local drift were in the age group of 25 ~ 29 years, which are − 0.611% per year and − 0.372% per year, respectively, and their local drift values are greater than their net drift values in the age group of 25 ~ 59 years; the peak local drift value of females was − 0.965% per year in the age group of 40 ~ 44 years, and their local drift values are greater than their net drift values in the age group of 25 ~ 59 years.

The longitudinal age curves of IHD mortality attributable to high LDL-C are shown in Fig. 3B. The longitudinal age curves of the overall, male and female IHD mortality attributable to high LDL-C showed a monotonic upward trend, and their IHD mortality attributable to the high LDL-C peak appeared in the 95 plus age group: 704.545 (95% CI:682.353 to 727.459), 692.722 (95% CI: 649.824 to 738.453) and 684.332 (95% CI:663.655 to 705.654), respectively. The IHD mortality attributable to high LDL-C in males was greater than that in females in all age groups.

The estimated period RR is presented in Fig. 3C. The period effect RR of overall, male and female IHD mortality attributable to high LDL-C showed a linear downward trend, and the risk of death gradually decreased over time. When 2002–2006 was used as a reference period (RR = 1), females presented higher RR values than did males before 2002, and females subsequently presented lower RR values than did males. The overall, male and female RR values in the period of 2002–2021 are all less than 1, and the lowest values appear in the period of 2017–2021, which were 0.791 (95% CI: 0.782 to 0.799), 0.809 (95% CI: 0.796 to 0.822) and 0.775 (95% CI: 0.766 to 0.783), respectively.

The estimated cohort RR is shown in Fig. 3D. After adjusting for age and period factors, the risk of overall, male and female IHD mortality attributable to high LDL-C gradually decreased. Taking 1957–1966 as the reference cohort (RR = 1), the RR values of females were greater than those of males before 1947–1956 and between 1962 – 1976. However, the RRs of males were greater than those of females between 1947 – 1957 and after 1972–1981. The overall, male and female RR values in the cohort after 1957–1966 were all less than 1, and the lowest values occurred from 1987–1996, with values of 0.771 (95% CI: 0.697 to 0.853), 0.821 (95% CI: 0.712 to 0.948) and 0.678 (95% CI: 0.602 to 0.763), respectively.

### Bayesian APC model prediction of IHD mortality attributable to high LDL-C

**Fig. 4:**
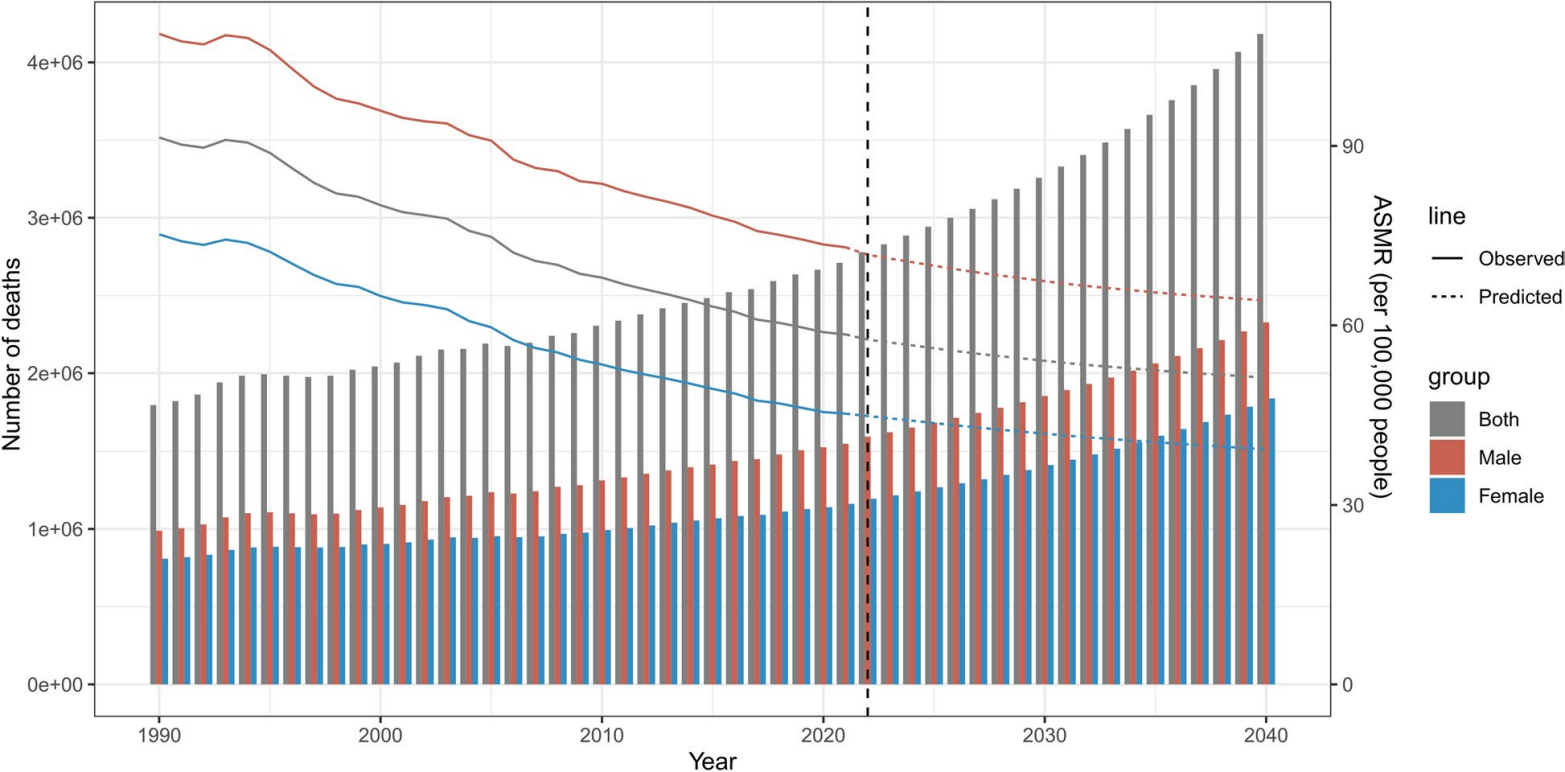
Prediction of the ASMR and number of deaths in IHD attributable to high LDL-C. IHD; ischemic heart disease, ASMR, age-standardized mortality rate; LDL-C, low-density lipoprotein cholesterol

The BAPC model predictions of the IHD ASMR attributable to high LDL-C are shown in Fig. 4. The BAPC model predicted that the overall, male and female ASMRs of IHD attributed to high LDL-C would significantly decrease from 2022 to 2040, and the ASMR of males will always be higher than that of females. The overall ASMR of IHD attributable to high LDL-C in 2030 and 2040 is predicted to be 54.058 per 100,000 people and 51.239 per 100,000 people. The female ASMR of IHD attributable to high LDL-C in 2030 and 2040 is predicted to be 41.939 per 100,000 people and 39.331 per 100,000 people. The male ASMR of IHD attributable to high LDL-C in 2030 and 2040 is predicted to be 67.376 per 100,000 people and 64.122 per 100,000 people.

The BAPC model predictions of IHD deaths attributable to high LDL-C are shown in Fig. 4. The BAPC model predicted that overall, male, and female IHD deaths attributed to high LDL-C would significantly increase from 2022 to 2040. The number of overall deaths in 2030 and 2040 is predicted to be 3.258 million and 4.183 million, respectively. The number of female deaths in 2030 and 2040 is predicted to reach 1.412 million and 1.840 million, respectively. The number of male deaths is much greater than that of female deaths, with 1.853 million and 2.327 million deaths in 2030 and 2040, respectively.

## Discussion

In 2021, the number of overall, male and female IHD deaths attributable to high LDL-C increased compared to 1990. However, after age standardization according to the standard population, the ASMR of IHD attributable to high LDL-C decreased, and the ASMR in males was greater than that in females. The joinpoint regression model revealed that overall, male and female AAPC were less than 0, and the APC model revealed that their net drift values were less than 0, both of which suggested that the overall, male, and female ASMR showed a downward trend from 1990–2021. The age‒period-cohort model results revealed that overall, male, and female IHD mortality attributable to high LDL-C all increased with age, and the period and cohort RR of mortality decreased with time. The BAPC model predicts that overall, male, and female ASMR will decrease in the next 19 years, but the number of deaths will continue to rise, indicating that the overall status of IHD mortality attributable to high LDL-C levels is not optimistic. A previous study revealed that a lack of insurance may lead to significant financial pressure on CVD patients and their families in low- and middle-income countries [16]. Low- and middle-income countries should invest more funds in the future to reduce the burden of cardiovascular disease, especially the IHD mortality burden attributable to high LDL-C. Therefore, the overall prevention and control of IHD mortality attributable to high LDL-C still has a long way to go.

The overall, male and female number of IHD deaths attributable to high LDL-C shown an upward trend, but their ASMR showed a downward trend. Similarly, it is predicted that the number of deaths will continue to increase in the next 19 years, but the ASMR will decrease. The inconsistent trend between the absolute number of deaths and the ASMR reveals the successful control of premature death, population growth, and aging processes. Our study revealed that the burden on males is greater than that on females because premenopausal females have lower LDL-C levels than males do because of estrogen [17]. Similar to a review [18] conducted in 2019, the Women's Ischemia Syndrome Evaluation (WISE) study revealed an alarming sevenfold increase in IHD risk for young females with endogenous estrogen deficiency. This correlates with a delay in the onset of IHD, which typically occurs 7–10 years later in women than in men in Western countries. Moreover, lower LDL-C also leads to lower IHD mortality rates. However, a review [18] revealed that females face more severe consequences from IHD and are less frequently subjected to interventional cardiac procedures than males, resulting in poorer health outcomes and even death. This disparity could be attributed to various factors, such as differences in symptom presentation, clinical characteristics, and even potential biases in healthcare delivery, as suggested by studies exploring sex differences in cardiac care [19]. In addition, the AAPC and net drift values of males are greater than those of females. Policymakers should pay attention to these gender differences and need to do more to reduce the burden on males. The results of the age effect analysis revealed that the overall risk of mortality increases with age. The mortality increases rapidly after the age group of 65–69, which is similar to previous research results [20]. LDL-C tends to decrease in both males and females older than 65 years [21]. The IHD mortality attributable to high LDL-C increases, which may be related to other risk factors for IHD in the elderly population. In addition to age itself being an important risk factor for IHD mortality, smoking and hypertension are important risk factors for IHD mortality in the elderly population [18]. As the global population ages, the prevention and control of IHD mortality attributable to high LDL-C levels will face greater challenges in the future. Therefore, the burden of IHD among the elderly population should receive attention.

The period effect analysis results revealed that the risk of death associated with overall, male, and female IHD attributable to high LDL-C decreased with time. Since 1990, the use of lipid-lowering drugs has greatly increased, while the average level of LDL-C has decreased [19]. Epidemiological data revealed that, among diabetic patients, lower LDL-C and increased use of lipid-lowering drugs explained 27.2% of the decline in IHD mortality between 1987–1996 and 2003–2009 [22]. Multiple studies have shown that lowering LDL-C is effective in reducing IHD mortality [23, 24]. In recent years, clinical guidelines have guided clinicians to strictly demand lowering LDL-C. A recent review revealed that CVD, including IHD, is a leading cause of mortality worldwide [25]. In addition to traditional statins, several new lipid-lowering drugs, such as cholesteryl ester-transfer protein inhibition and proprotein convertase subtilisin/kexin type 9, have been recommended [26–30], which have to some extent reduced the mortality rate of IHD. Furthermore, potential factors such as advancements in medical technology, changes in lifestyle, and increased awareness of IHD are also important factors contributing to the decline in IHD mortality.

The cohort effect analysis results revealed that the overall, male, and female IHD risk attributable to high LDL-C decreased with the passage of the birth cohort, with the highest risk of death occurring in the birth cohort from 1992–1996. On the one hand, the younger generation is increasingly educated, paying more attention to physical health and receiving better medical resources [30]. On the other hand, with the transformation of information dissemination methods, especially the rapid development of the internet in recent years, people have received more comprehensive health knowledge, improved awareness of disease prevention and control, and gradually improved their level of health literacy.

### Strengths and limitations

The strengths of this study lie in its use of extensive Global Burden of Disease data, providing a comprehensive analysis of IHD mortality trends attributable to high LDL-C from 1990 to 2021. The application of sophisticated statistical models, such as joinpoint regression and age-period-cohort analysis, offers detailed insights into the dynamics of these trends over time. Moreover, the integration of Bayesian predictive modeling enhances the foresight of future mortality patterns. However, like all studies, ours is subject to certain limitations. The GBD database relies on various data sources with inconsistent collection standards, potentially affecting the study's reliability. The analysis is global and lacks detailed regional and national insights. Ecological fallacies may skew the application of findings to individuals, necessitating validation through large-scale cohort studies. The BAPC model's reliance on prior distributions can impact prediction accuracy and may overlook rapid or localized health data changes. The study does not isolate high LDL-C effects from other risk factors and omits recent findings linking nonalcoholic fatty liver disease [31] and sarcopenia [32] to IHD.

## Conclusion

IHD mortality attributable to high LDL-C is a critical health challenge that notably impacts elderly individuals and males and is likely to impose a significant healthcare burden. Comprehensive strategies are necessary, with a focus on early detection, public awareness, and lifestyle interventions. Ensuring pharmaceutical access and supportive health policies is crucial. Community engagement and robust cardiovascular care infrastructure, along with research on new treatments and a rigorous evaluation framework, are essential to mitigate the impact of high LDL-C levels on IHD mortality and enhance public health.

## Data Availability

The data in this study were obtained from the GBD 2021 Global Health Data Exchange website. As a derivative assessment of preexisting data, there is no need for supplementary ethical evaluation or informed consent from human subjects.
